# Enhancement of agri-food by-products: green extractions of bioactive molecules with fungicidal action against mycotoxigenic fungi and their mycotoxins

**DOI:** 10.3389/fnut.2023.1196812

**Published:** 2023-05-25

**Authors:** Paola Giorni, Giulia Bulla, Giulia Leni, Mariangela Soldano, Massimo Tacchini, Alessandra Guerrini, Gianni Sacchetti, Terenzio Bertuzzi

**Affiliations:** ^1^Dipartimento delle Produzioni Vegetali Sostenibili (DIPROVES), Facoltà di Scienze Agrarie, Alimentari e Ambientali, Università Cattolica del Sacro Cuore, Piacenza, Italy; ^2^Dipartimento di Scienze Animali, della Nutrizione e degli Alimenti (DIANA), Facoltà di Scienze Agrarie, Alimentari e Ambientali, Università Cattolica del Sacro Cuore, Piacenza, Italy; ^3^Centro Ricerche Produzioni Animali Soc. Cons. p.A, Reggio Emilia, Italy; ^4^Dipartimento di Scienze della Vita e Biotecnologie, Università Degli Studi Di Ferrara, Ferrara, Italy

**Keywords:** food waste, plant extract, aflatoxins, fumonisins, deoxynivalenol, ochratoxin A, *Alternaria* toxins

## Abstract

**Introduction:**

Today, alternative strategies based on the use of bioactive compounds have been proposed to reduce mycotoxin contamination and limit the use of chemical fungicides.

**Methods:**

In the present work, several by-products collected from the agri-food chain (i.e., red and white grape marc, red grapevine leaves, grape seeds and stalks, pear, apple, green beans, tomato, and spent hops) were subjected to green extraction protocols (i.e., steam distillation, Ultrasound-Assisted, and Naviglio® extraction) to obtain extracts rich in polyphenols and terpenes. Each extract was assessed *in vitro* for its ability to inhibit the development of the main mycotoxigenic species and related mycotoxins.

**Results and Discussion:**

*Aspergillus flavus* and *A. carbonarius* were significantly reduced by pear (from −45 to −47%) and grape marc (from −21 to −51%) extracts, while *F. graminearum* was shown to be highly influenced by grape stalk, pear, and grape marc extracts (−24% on average). On the contrary, *F. verticillioides* was inhibited only by pear (−18%) and to a very low and negligible extent by apple (−1%) and green beans (−3%). Regarding the reduction of mycotoxins, the extracts were able to inhibit OTA from 2 to 57%, AFB1 from 5 to 75%, and DON from 14 to 72%. The highest percentages of reduction were obtained against FBs (from 11 to 94%), ZEN (from 17 to 100%), and *Alternaria* toxins (from 7 to 96%). In conclusion, this work provided promising results for the production of bioactive extracts obtained from agri-food by-products, which could be exploited as potential biofungicides against the development of mycotoxigenic fungi and related mycotoxins.

## 1. Introduction

The presence of mycotoxins in food commodities is a concrete risk worldwide, with important potential impacts on human and animal health; therefore, their presence in agricultural products is the subject of extensive research across the globe. Cereals, dried fruits, and spices may be exposed to contamination by several mycotoxigenic fungal species and related mycotoxins (1–5). Moreover, in a climate change scenario where high temperatures and extreme events such as floods or droughts are reached, the development of mycotoxigenic fungi could find suitable environmental conditions for their easy growth. The European Union (EU) has established maximum levels for the major mycotoxins (6, 7). Recently, the European Commission also published maximum levels for *Claviceps* ergot and a recommendation for *Alternaria* toxins in food products, underscoring the increasing concern about the possible effects of these mycotoxins on human health (8, 9).

Preventive methods, such as good agricultural practices and the use of appropriate chemical products, are considered the best choices to reduce mycotoxin contamination. However, the increasing interest of consumers in the adverse health effects presumably produced by pesticides has led to a growing demand for natural products, as their use is seen as an improvement in food quality and consumer safety. Due to the increasing need to reduce the use of chemical pesticides in agriculture, also according to the guidelines of the EU Green Deal, and to achieve both the environmental emergency and the consumer demand for safer agro-food products, several bioactive compounds showed to be promising alternatives as inhibitors of antifungal activity and mycotoxin production.

In *in vitro* studies, phenolic compounds, which are naturally found in plants, herbs, fruits, and other vegetables, showed antifungal activity and could limit mycotoxin production (10–12). Polyphenols were able to inhibit trichothecene-producing *Fusarium* (13), isoflavones showed weak inhibitory activity against *Fusarium graminearum* (14), and several essential oils were efficient in mitigating mycotoxigenic *Aspergillus* and *Penicillium* species (15, 16). In general, naturally occurring phenolic compounds and terpenes seem to be really effective as anti-mycotoxin agents. Food waste represents a reservoir of these bioactive compounds, and their valorization through appropriate biorefinery approaches could play a crucial role in sustainable and zero-waste global development. In fact, today, the residual biomass discarded by the whole food chain amounts to 1,300 Mtons/year, with a significant impact on the ecosystem. For this reason, the circular economy is now being explored as a new economic system that allows the reuse and valorization of waste and by-products as a resource to manufacture new materials and products. In this perspective, food waste extracts could be a potential source of bioactive polyphenols that can be exploited for many different purposes (e.g., food additives, preservatives, coloring agents, and nutraceuticals) (17). Besides their common application as antioxidants and antimicrobials, they could also be used as biofungicides and inhibit mycotoxin production in different raw commodities (18). The by-products considered come from three different agri-food supply chains (wine, canning, and brewing) in the Emilia Romagna region, where the two collaborating universities for this study are located. The most abundant by-products of the wine and canning supply chains were considered: marc, stalks, grape seeds, and leaves from the wine supply chain; and pear, apple, green bean, and tomato processing waste from the canning supply chain. In the case of the brewing chain, the spent hops were considered for their essential oil content and anti-fungal activity.

Many different methodologies can be used for bioactive compounds, ranging from conventional ones such as acid and alkaline hydrolysis, solvent extraction, and Soxhlet extraction to more sustainable and new techniques that are more cost-effective and highly efficient (19). The extraction techniques used for this study (Ultrasound-Assisted Extraction, Naviglio®, and steam distillation) were chosen based on secondary metabolites with possible antifungal activity against the microorganisms able to be obtained and also for their low environmental impact. The first two, carried out with hydroalcoholic solvents, were necessary for the extraction of polar molecules, while steam distillation, the method of choice for obtaining essential oils (also listed in the Official European Pharmacopoeia for numerous drugs), was used to obtain hop essential oil.

Using these extraction processes, different agri-food wastes have been exploited for their biological, biostatic, and biocidal activities and have been considered for their possible use as alternatives to protect plants from contamination by fungi and mycotoxins, limiting the use of chemical fungicides.

## 2. Materials and methods

### 2.1. Chemicals and raw materials

All of the solvents, standards, salts, acids, and bases were of analytical grade and were purchased from Sigma-Aldrich-Merck (Darmstadt, Germany) or Carlo Erba (Milan, Italy). Aflatoxin B1 (AFB_1_), ochratoxin (OTA), fumonisins B1 and B2 (FB_1_, FB_2_), deoxynivalenol (DON), zearalenone (ZEN), alternariol (AOH), alternariol monomethyl ether (AME), and tenuazonic acid (TEA) were purchased from Sigma-Aldrich (St. Louis, MO, USA). The by-products selected for this study were: red and white grape marc (RG and WG, respectively); red grapevine leaves (RGL); grape seeds (VIN) and stalks (GS); pear (PE), apple (AP); green beans (GB); tomato (TO); and spent hops (HW).

### 2.2. Extractions from food waste

#### 2.2.1. Ultrasound-assisted extraction

Ultrasound-assisted extraction (UAE) was performed in an ultrasonic bath (Ultrasonik 104X, Ney Dental International, MEDWOW, Cyprus) at a working frequency of 48 kHz. An aliquot of 50 g of each sample was extracted with 650 ml of a 50% ethanolic solution for 80 min at room temperature (solvent/solid ratio of 13 ml/g of dried matrix). Each aliquot was subjected to a triple extraction, each time with fresh solvent, to increase the yield of the procedure. The extracts were then filtered and lyophilized. Each extraction was performed in triplicate.

#### 2.2.2. Naviglio® extraction (NAV)

The Naviglio® extractor (Atlas Filtri, Italy) was used to extract solids using a pressurized solvent extraction method (20). Briefly, 30 g of each sample was placed in a bag with a 60 μm filtering membrane and transferred to the chamber of the Naviglio extractor; 400 ml of a 50% ethanolic solution was added. The extraction process consisted of 10 cycles, each divided into two phases, one static, and one dynamic: the former was set for 5 min, while the dynamic phase was set for 3 min, for a total extraction time of 80 min. The extracts were then filtered and lyophilized. Each extraction was performed in triplicate.

#### 2.2.3. Steam distillation of spent hops

An aliquot of 300 g of the spent hops was used to obtain essential oils by 3 h steam distillation (DIS) with a Clevenger apparatus according to the methods of the European Pharmacopoeia. The extract yield was determined on a volume-to-wet-weight basis, and the samples, dehydrated, were stored in glass vials with Teflon sealed caps at −18 ± 0.5°C in the absence of light until analysis.

### 2.3. Extract characterization

#### 2.3.1. Determination of total phenolic content, total flavonoid content, and total proanthocyanidin content

The determination of total polyphenolic, flavonoid, and proanthocyanidin content was performed using a Thermo Spectronic Helios-γ spectrophotometer (Gebraucht, Germany) according to the method described by Greco et al. (21). The total phenolic content (TPC) results are expressed as the g equivalent of gallic acid/100 g of dried extract, total flavonoid content (TFC) as the g equivalent of hyperoside/100 g of dried extract, and total proanthocyanidin content (TPrC) as the g equivalent of cyanidin chloride/100 g of dried extract. Each experiment was performed in triplicate.

#### 2.3.2. RP-HPLC-DAD analyses

RP-HPLC analyses were performed with a JASCO modular HPLC system (Tokyo, Japan, model PU 2089) coupled to a diode array apparatus (MD 2010 Plus). The HPLC was equipped with an injection valve with a 20 μl sampling loop and an Eclipse-PLUS-C18 column (25 mm × 0.46 cm, 5 μm; Phenomenex, Bologna, Italy) at a flow rate of 1.0 ml/min. The identification and quantification of different polyphenols in the extracts of agri-food waste were performed following the experimental conditions also used by Tacchini et al. (caftaric acid and flavonoids) (22), Bernardi et al. (anthocyanins) (23), and Kammerer et al. (gallic acid and catechins) (24). The different peaks were identified by comparing their UV spectra and retention times with those of pure standards. Dedicated JASCO software (ChromNAV version 2.02.01) was used to calculate peak areas by integration. Each experiment was performed in triplicate.

#### 2.3.3. GC–MS and GC-FID analyses

The samples obtained from steam distillation were diluted, and 1 μl of the solution was injected for gas chromatography (GC). GC analysis was performed on a Varian GC3800 gas chromatograph equipped with a Varian MS-4000 mass spectrometer using electron impact (EI) and hooked to the NIST library, and a ThermoQuest GC-Trace (ThermoQuest Italia, Rodano, Italy) coupled to a flame ion detector. The operating conditions are the same as those reported by Tacchini et al. (25). Each experiment was performed in triplicate.

### 2.4. Preparation of fungal strains

Representative mycotoxigenic fungal strains were obtained from official fungal collections, as reported in Table 1.

**Table 1:** Mycotoxigenic fungal strains obtained from official collections and used in the experiments.

Fungal species	Code number	Official collection
*Aspergillus flavus*	ITEM 8069	ISPA-CNR BARI
*Aspergillus carbonarius*	ITEM 5012	ISPA-CNR BARI
*Fusarium verticillioides*	ITEM 10027	ISPA-CNR BARI
*Fusarium graminearum*	ITEM 646	ISPA-CNR BARI
*Alternaria alternata*	CBS 118814	Westerdijk Fungal Biodiversity Institute

Fungal strains were centrally transferred on Petri dishes (Ø 9 cm) containing potato dextrose agar (PDA, Biolife, Milan, Italy) and incubated at 25°C for 7 days (12 h light/12 h dark photoperiod). After the incubation period, the developed fungal colonies were used as a source for further inoculations.

### 2.5. Extract evaluation – *in vitro* test

All the extracts were tested for their ability to reduce mycotoxigenic fungal growth and mycotoxin production at a concentration of 1,000 mg/L. On Petri dishes (Ø 9 cm) containing potato dextrose agar (PDA, Biolife, Milan, Italy), 1 mL of the extract solution was distributed with a sterile spreader. Fungal inoculation was performed by cutting agar discs (Ø 2 mm) using a sterile cork borer from the edge of the fungal colony prepared as previously described and putting them at the center of the dish. Petri dishes centrally inoculated with fungi but without the addition of extracts were considered untreated. Inoculation was performed for the fungal species listed in Table 1, and the experiment was performed in triplicate.

Petri dishes were incubated at 25°C for 14 days. At the end of the incubation period, fungal growth and mycotoxin production were determined.

#### 2.5.1. Measurement of fungal growth

The diameter of the fungal colonies was measured along two perpendicular diagonals crossing the inoculum point. The percentage reduction in fungal growth in the presence of the extracts was calculated by comparing the fungal growth diameters obtained in untreated dishes with the fungal growth diameters obtained in the presence of each extract (26).

#### 2.5.2. Mycotoxin production

The entire contents of each Petri dish were homogenized and extracted with 40 mL of CH3CN using a rotary shaker for 45 min. After centrifugation and dilution (1 + 9 v/v) of the extract, mycotoxins were quantified using instrumental methods previously developed and published in our laboratory (27–31).

### 2.6. Data analyses

Data were transformed prior to statistical analysis; specifically, data on fungal growth reduction were arcsine transformed, while data on mycotoxin production (values +1) were logarithmically transformed (32).

Analysis of variance (ANOVA) was calculated using the generalized linear model (GLM) procedure of the statistical IBM SPSS Statistics 27 package (IBM Corp., Armonk, NY, United States), while significant differences were highlighted using the Tukey’s test (*p* ≤ 0.05) for mean separation.

Also for mycotoxins, the percentage reduction of the fungal ability to produce its own mycotoxin was calculated by comparing the mycotoxin production in untreated dishes with the mycotoxin production obtained in the presence of each extract.

## 3. Results

### 3.1. Characterization of the extracts

A total of 14 different extracts were obtained from 10 different food by-products using the different high-tech methodologies described previously. Extracts obtained using Ultrasound-Assisted Extraction (UAE) and pressurized liquid extraction with Naviglio® technology (NAV) were primarily analyzed for the content of polyphenolic compounds (Table 2).

**Table 2:** Yield and total phenolic compounds (TPC), flavonoids (TFC), and proanthocyanidins (TPrC) content in agri-food extracts.

Extract (Abbreviation)	Yield %	TPC (g GAE/100 g dried extract)	TFC (g Hyp/100 g dried extract)	TPrC (g Cyanidin Chloride/100 g dried extract)
Red Grape marc UAE (RG UAE)	23.26 ± 0.30	259.20 ± 8.47	13.59 ± 1.10	89.70 ± 5.44
Red Grape marc NAV (RG NAV)	11.29 ± 0.64	205.59 ± 7.75	21.88 ± 1.32	61.76 ± 6.72
White Grape marc UAE (WG UAE)	30.55 ± 0.71	197.26 ± 5.32	4.40 ± 0.24	31.36 ± 1.38
White Grape marc NAV (WG NAV)	14.26 ± 0.92	165.43 ± 20.73	10.85 ± 1.28	33.50 ± 3.21
Red Grapevine Leaves (RGL UAE)	15.27 ± 0.72	253.19 ± 11.41	113.48 ± 3.24	10.02 ± 0.18
Red Grapevine Leaves (RGL NAV)	11.75 ± 0.84	148.21 ± 1.10	126.41 ± 10.25	3.54 ± 0.18
Grape seeds UAE (VIN)	9.32 ± 0.48	514.26 ± 5.70	60.56 ± 3.51	92.17 ± 7.10
Grape stalks UAE (GS)	31.72 ± 0.54	198.95 ± 10.40	22.24 ± 0.51	9.14 ± 0.09
Pear (PE)	24.34 ± 0.22	105.94 ± 9.84	17.96 ± 1.25	3.56 ± 0.11
Apple (AP)	34.07 ± 2.33	29.50 ± 1.53	3.88 ± 0.33	0.80 ± 0.08
Green Bean (GB)	37.92 ± 1.83	41.17 ± 1.11	8.99 ± 0.63	0.96 ± 0.03
Tomato (TO)	15.97 ± 0.82	17.56 ± 0.55	7.33 ± 2.06	0.58 ± 0.04
Spent Hops UAE (HW)	22.83 ± 0.03	18.84 ± 0.32	15.06 ± 1.33	1.26 ± 0.02

TPC, total phenolic content; TFC, total flavonoid content; TPrC, total proanthocyanidin content; GAE, gallic acid equivalent; Hyp, hyperoside.

The extraction methods used for the production of extracts allowed yields ranging from 9.32% in VIN to 37.92% in GB. Red grape marc, white grape marc, and grapevine leaves were extracted using both NAV and UAE techniques, with UAE providing the highest extract yields. These extracts were also subjected to chemical analysis in order to quantify the content of total phenols, flavonoids, and proanthocyanidins. Total phenolic compounds ranged from 17.56 g gallic acid equivalent/100 g dried extract in TO to 514.26 g gallic acid equivalent/100 g dried extract in VIN. As previously reported, for those samples that were subjected to both UAE and NAV extraction techniques, UAE allowed the recovery of the highest amount of phenolic compounds. Total flavonoids were determined in concentrations ranging from 3.88 g of hyperoside/100 g dried extract in AP to 126.41 g of hyperoside/100 g dried extract in RGL NAV; the highest amount of total proanthocyanidins was detected in VIN extract (92.17 g cyanidin chloride/100 g dried extract), while the lowest was TO (0.58 g cyanidin chloride /100 g dried extract). Comparing these results, it can be observed that, while UAE gave a higher extraction yield and a higher quantity of total phenolic content than NAV, the quantification of total flavonoids shows the opposite trend, explaining a possible selectivity of this technique towards the latter molecular category. The first eight extracts in Table 2 came from waste from the wine supply chain and showed a higher amount of total phenols compared to the other by-products selected in the current study. In particular, among all the extracts, grape seed UAE was characterized by the highest content of total phenols (514.26 g gallic acid equivalent /100 g dry extract), composed of 18% proanthocyanidins and 12% flavonoids. This matrix, although of high commercial value, showed the lowest extractive yield compared to the other agri-food extracts obtained. Considering the quantification of total flavonoids, RGL extracted using the NAV technique was the richest waste matrix among those analyzed, showing twice the flavonoid content of grape seeds. The flavonoid fraction of this matrix was then studied in detail, and individual flavonoids were identified and quantified. Table 3 shows the results of the flavonoid quantification of the leaf extract.

**Table 3:** Main flavonoid content in grapevine leaf extracts (RGL) obtained by both ultrasound-assisted extraction (UAE) and Naviglio extraction (NAV).

Extract	Caftaric acid	Quercetin-3-*O*-glucuronide and Quercetin-3-*O*-glucopyranoside	Q-3-*O*-rutinoside	k-3-*O*-glucoside	k-3-*O*-rutinoside
	mg/g extract ± SD	mg/g extract ± SD	mg/g extract ± SD	mg/g extract ± SD	mg/g extract ± SD
RGL UAE	5.35 ± 0.46	36.53 ± 0.87	1.41 ± 0.11	2.32 ± 0.13	1.41 ± 0.23
RGL NAV	2.68 ± 0.23	47.21 ± 0.45	2.75 ± 0.05	5.26 ± 0.03	2.96 ± 0.06

Q-3-*O*-rutinoside: quercetin-3-*O*-rutinoside; k-3-*O*-glucoside: kaempferol-3-*O*-glucoside; k-3-*O*-rutinoside: kaempferol-3-*O*-rutinoside.

In this case, too, the NAV technique gave the best quantitative results, highlighting the main flavonoids as quercetin and kaempferol derivates.

Another molecular category of interest from the point of view of reducing fungal growth is anthocyanins (33); a preliminary quantification was carried out in the red pomace extracts (Table 4).

**Table 4:** Main anthocyanins in grape pomace extract (RG) obtained by both ultrasound-assisted extraction (UAE) and Naviglio extraction (NAV).

	Kuromanin	Myrtillin	Oenin
	mg/g extract ± SD	mg/g extract ± SD	mg/g extract ± SD
RG UAE	0.24 ± 0.01	1.01 ± 0.10	4.14 ± 0.12
RG NAV	0.17 ± 0.01	1.07 ± 0.05	3.22 ± 0.14

NAV and UAE did not significantly influence the concentration of anthocyanins detected in the RG extracts, and oenin was the most abundant in both samples.

Moreover, chlorogenic acid, known for its antifungal action (34), was determined in AP and PE extracts since it is one of the main secondary metabolites identified in these vegetables. Pear processing residues obtained with UAE showed a chlorogenic acid content of 2.54 ± 0.21 mg/g of extract, while apple processing residues obtained with UAE showed 0.70 ± 0.03 mg/g of extract.

Finally, the HW extract (from the IPA mixture after brewing) was obtained by hydro-distillation and characterized for its terpene content by GC–MS and GC-FID analysis (Table 5).

**Table 5:** GC-MS-FID chemical characterization of spent hop distillation extract.

*n*°	Compound^a^	Area %^b^			AI exp.^c^	AI lit^c^
1	Beta-pinene	0.49	±	0.02	971	979
2	Myrcene	29.13	±	1.45	986	991
3	Propanoic acid, 2-methyl-, 3-methylbutylester	0.13	±	0.01	1010	1009
4	Propanoic acid, 2-methyl-, 2-methylbutylester	0.23	±	0.01	1013	1014
5	Limonene	0.32	±	0.01	1023	1029
6	2-Undecanone	0.91	±	0.03	1292	1294
7	*Cis*-pinocarvyl acetate	0.61	±	0.02	1309	1311
8	Methyl geranate	0.63	±	0.02	1325	1325
9	Alpha-ylangene	0.15	±	0.01	1367	1375
10	Alpha-copaene	0.52	±	0.02	1373	1377
11	*n*-Tetradecane	0.16	±	0.01	1396	1400
12	Beta-caryophyllene	13.90	±	0.58	1406	1409
13	Beta-copaene	0.55	±	0.03	1420	1432
14	Alpha-caryophyllene	29.07	±	1.05	1448	1455
15	γ-Gurjunene	1.62	±	0.06	1470	1477
16	Geranyl propanoate	0.21	±	0.01	1474	1478
17	Beta-selinene	0.68	±	0.02	1481	1490
18	Cubenene	0.42	±	0.01	1486	1496
19	Alpha-selinene	0.65	±	0.03	1487	1498
20	Gamma-muurolene	0.16	±	0.01	1490	1499
21	Alpha-muurolene	0.34	±	0.01	1492	1500
22	2-Tridecanone	1.22	±	0.05	1495	1495
23	Gamma-cadinene	2.37	±	0.10	1505	1514
24	Geranyl isobutyrate	0.70	±	0.03	1507	1515
25	Delta-cadinene	2.78	±	0.12	1512	1523
26	*Trans*-calamene	0.40	±	0.02	1516	1529
27	Alpha-cadinene	0.83	±	0.04	1530	1539
28	Selina-3,7(11)-diene	0.52	±	0.02	1535	1547
29	*n*-Tridecanol	0.23	±	0.01	1571	1572
30	Caryophyllene oxide	1.46	±	0.05	1577	1583
31	Cedrol	0.24	±	0.01	1594	1600
32	Geranyl 2-methyl butanoate	0.19	±	0.01	1597	1601
33	Humulene epoxide II	2.43	±	0.12	1605	1608
34	1-epi-Cubenol	0.13	±	0.01	1626	1629
35	Eremoligenol	0.22	±	0.01	1629	1631
36	Alpha-acorenol	0.28	±	0.01	1632	1633
37	Alpha-muurolol	0.26	±	0.01	1641	1646
38	Alpha-cadinol	0.27	±	0.01	1656	1654
39	Selin-11-en-4-alpha-ol	2.95	±	0.14	1660	1660
40	14-hydroxy-9-epi-(*E*)-caryophyllene	0.20	±	0.01	1665	1667
41	Epi-beta-bisabolol	0.94	±	0.03	1670	1672
42	2-Pentadecanone	0.30	±	0.03	1698	1697
	Total	99.39				

Major compounds are indicated in bold. ^a^Compounds are listed in order of elution, and their nomenclature is according to the NIST (National Institute of Standards and Technology) library. ^b^Relative peak areas calculated by GC-FID. ^c^AI exp: arithmetic retention indices calculated on Varian VF-5 ms columns for comparison with AI lit: arithmetic retention indices (35).

The extraction yield of the exhausted hop (Hop-EO) after IPA brewing was 0.10%, and the most abundant terpenes were: myrcene (29.1%), *α*-caryophyllene (29.1%), *β*-caryophyllene (13.9%), cadinene (5.0%), and humulene epoxide (2.5%).

### 3.2. Inhibition of mycotoxigenic fungal growth

**Figure 1:**
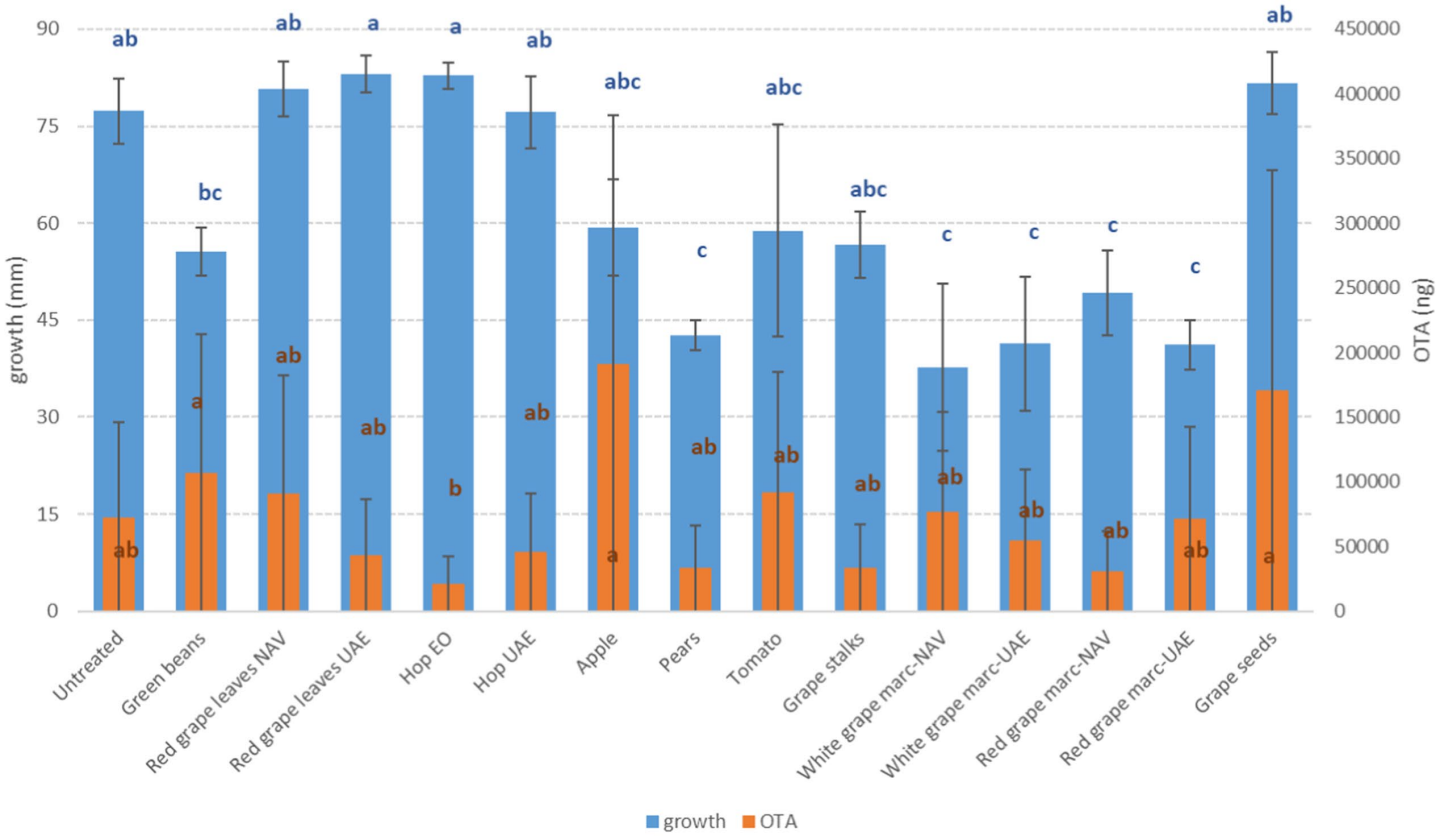
Mean development (mm) and ochratoxin A production (OTA, ng) of *A. carbonarius* grown on Petri dishes (Ø 90 mm) with potato dextrose agar and 1 ml of different extracts obtained from agricultural production waste at a concentration of 1,000 mg/L after 14 days of incubation at 25°C. Different letters mean significant differences according to Tukey’s test; blue letters refer to significant differences between the theses related to fungal growth, while orange letters refer to significant differences between the theses related to mycotoxin production.

**Figure 2:**
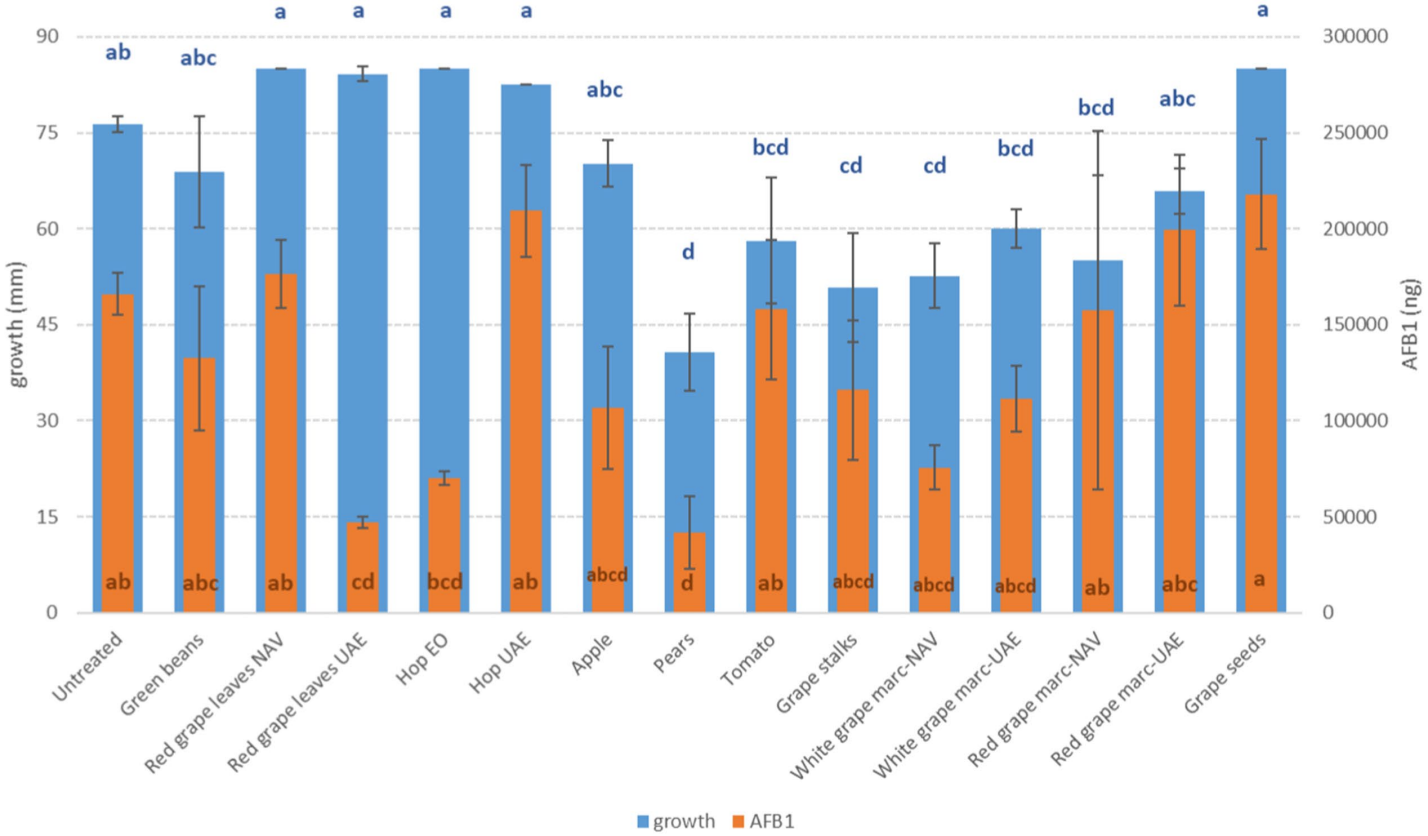
Mean development (mm) and aflatoxin B1 production (AFB1, ng) of *A. flavus* grown on Petri dishes (Ø 90 mm) with potato dextrose agar and 1 ml of different extracts obtained from agricultural production waste at a concentration of 1,000 mg/L after 14 days of incubation at 25°C. Different letters mean significant differences according to Tukey’s test; blue letters refer to significant differences between the theses related to fungal growth, while orange letters refer to significant differences between the theses related to mycotoxin production.

The mycotoxigenic fungi tested were shown to be differentially affected by the extracts. In particular, *A. flavus* and *A. alternata* were the fungi inhibited by the highest (*n* = 9) and the lowest (*n* = 1) number of extracts, respectively (Table 6). In general, the growth of the *Aspergillus* species considered in this study (*A. flavus* and *A. carbonarius*) was more affected by PE (reductions from 45 to 47%) and RG-WG (reductions from 21 to 51%) extracts, but also TO (−24%) and GS (reductions from 27 to 33%) extracts, which proved to be good inhibitors for these fungi’s development. For *Aspergillus* species, differences were found for AP and GB extracts; in fact, in both cases, *A. carbonarius* was more affected by their presence (reduction of 23 and 28%, respectively) (Figure 1) when compared with *A. flavus* (reduction of 8 and 10%, respectively) (Figure 2).

**Table 6:** Average percentage reduction or increase in fungal growth and associated mycotoxins following treatment with agri-food extracts.

	Apple	Grape seeds	Grape stalks	Green beans	Hop IPA	Hop IPA UAE	Pear	Red grape leaves UAE	Red grape leaves NAV	Tomato	WG-NAV	WG-UAE	RG-NAV	RG-UAE
*F. graminearum*														
Growth	0.0	0.0	−22.5 ± 18.0	0.0	0.0	0.0	−22.8 ± 12.8	0.0	0.0	0.0	−14.7 ± 14.5	−25.0 ± 2.9	−14.7 ± 5.1	−7.8 ± 7.4
DON	−49.3 ± 9.8	−11.7 ± 28.6	−8.8 ± 67.1	−38.5 ± 20.2	−41.6 ± 26.8	5.5 ± 26.3	56.9 ± 78.9	−46.4 ± 12.3	−8.5 ± 22.6	−70.4 ± 11.4	58.2 ± 188	−79.7 ± 5.4	116 ± 249	48.0 ± 141
ZEN	−85.4 ± 18.5	−90.0 ± 7.8	−97.4 ± 3.4	−75.5 ± 32.1	−89.3 ± 10.6	−97.0 ± 2.8	−100	−81.0 ± 13.3	−63.0 ± 37.6	−94.7 ± 5.6	−100	−100	−100	−97.6 ± 3.5
*F. verticillioides*														
Growth	−1.2 ± 2.2	9.1 ± 0.1	5.0 ± 3.9	−2.5 ± 4.8	9.1 ± 0.1	8.7 ± 0.7	−18.3 ± 6.9	7.4 ± 3.0	9.1 ± 0.1	9.1 ± 0.1	2.6 ± 3.7	4.6 ± 1.7	3.3 ± 5.1	5.2 ± 0.8
FBs (FB_1_ + FB_2_)	14.0 ± 25.4	−10.8 ± 39.3	−75.8 ± 14.3	−63.8 ± 19.0	26.4 ± 46.9	−29.8 ± 24.1	−81.4 ± 5.3	25.7 ± 23.1	−52.0 ± 48.9	−24.4 ± 19.5	−94.0 ± 0.1	−71.0 ± 4.0	−49.0 ± 5.7	−76.6 ± 9.6
*A. flavus*														
Growth	−8.1 ± 5.9	11.4 ± 0.1	−33.4 ± 13.6	−9.8 ± 13.9	11.4 ± 0.1	8.1 ± 0.1	−46.7 ± 9.7	10.3 ± 1.9	11.4 ± 0.1	−23.8 ± 15.7	−31.0 ± 8.2	−21.4 ± 4.9	−27.9 ± 21.5	−13.8 ± 5.8
AFB_1_	−35.8 ± 23.5	31.4 ± 21.1	−30.1 ± 26.9	−20.0 ± 27.7	−57.7 ± 2.7	26.3 ± 17.7	−74.8 ± 13.9	−71.5 ± 2.2	6.2 ± 13.1	−4.8 ± 26.7	−54.4 ± 8.4	−32.8 ± 12.5	−5.0 ± 69.1	20.1 ± 29.1
*A. carbonarius*														
Growth	−23.3 ± 11.9	5.6 ± 7.5	−26.7 ± 8.1	−28.0 ± 5.9	7.1 ± 3.3	−0.2 ± 8.8	−44.8 ± 3.6	7.3 ± 4.5	4.5 ± 6.7	−23.9 ± 26.0	−51.3 ± 20.6	−46.6 ± 16.4	−36.4 ± 10.4	−46.8 ± 6.0
OTA	164 ± 86.6	134 ± 70.6	−53.9 ± 25.8	47.1 ± 39.3	−70.7 ± 40.6	−37.4 ± 56.8	−54.3 ± 21.3	−40.2 ± 16.0	25.6 ± 19.7	26.9 ± 82.7	6.1 ± 87.2	−24.9 ± 50.6	−57.4 ± 11.2	−2.3 ± 23.4
*A. alternata*														
Growth	0.4 ± 10.8	22.9 ± 5.1	23.6 ± 1.3	−5.8 ± 8.6	10.0 ± 7.3	15.7 ± 8.0	15.7 ± 6.7	13.2 ± 21.8	15.0 ± 2.6	9.5 ± 6.8	25.8 ± 0.1	22.4 ± 6.0	18.9 ± 6.2	17.4 ± 6.7
TeA	33.6 ± 23.0	−50.3 ± 10.0	15.4 ± 20.0	−9.7 ± 8.4	−26.0 ± 30.6	−42.8 ± 9.1	−6.6 ± 7.4	−12.1 ± 18.7	−48.4 ± 2.3	17.7 ± 37.8	21.7 ± 30.7	13.4 ± 6.7	34.6 ± 27.3	−46.6 ± 5.3
AOH	31.3 ± 46.7	−17.1 ± 19.0	−61.5 ± 7.8	−61.8 ± 41.1	52.5 ± 96.7	−10.7 ± 39.7	−77.8 ± 14.7	−20.8 ± 53.7	4.7 ± 14.9	−20.0 ± 86.9	−82.8 ± 13.4	−82.3 ± 13.9	−77.9 ± 11.1	−17.3 ± 30.8
AME	−26.1 ± 9.6	−45.2 ± 21.9	−83.7 ± 0.3	−94.4 ± 6.6	15.3 ± 82.4	−23.7 ± 40.1	−90.9 ± 8.4	−58.5 ± 32.7	−34.0 ± 30.6	−69.5 ± 41.4	−95.9 ± 3.2	−93.1 ± 5.7	−89.5 ± 3.2	−48.5 ± 28.7

**Figure 3:**
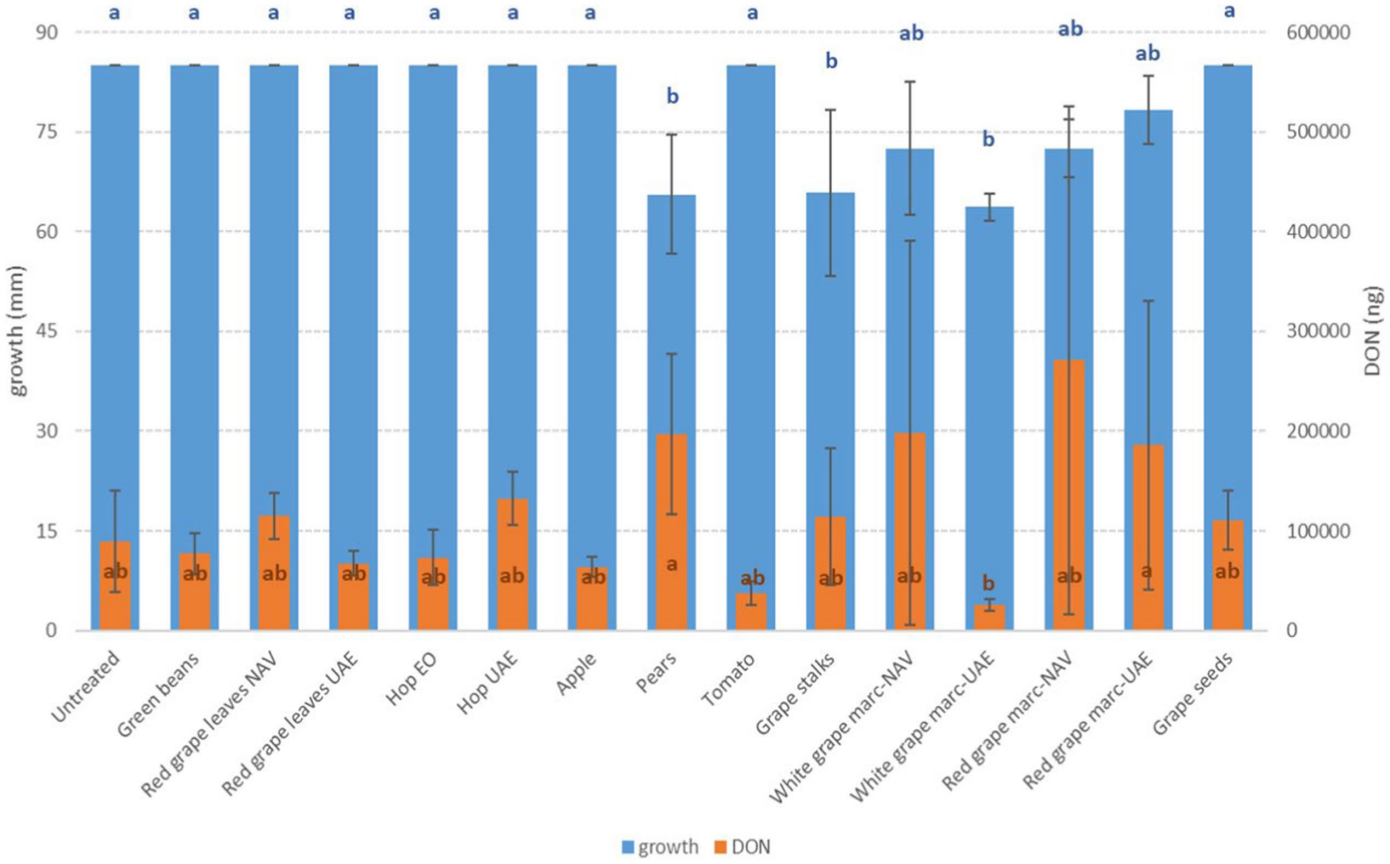
Mean development (mm) and deoxynivalenol production (DON, ng) of *F. graminearum* grown on Petri dishes (Ø 90 mm) with potato dextrose agar and 1 ml of different extracts obtained from agricultural production waste at a concentration of 1,000 mg/L after 14 days of incubation at 25°C. Different letters mean significant differences according to Tukey’s test; blue letters refer to significant differences between the theses related to fungal growth, while orange letters refer to significant differences between the theses related to mycotoxin production.

**Figure 4:**
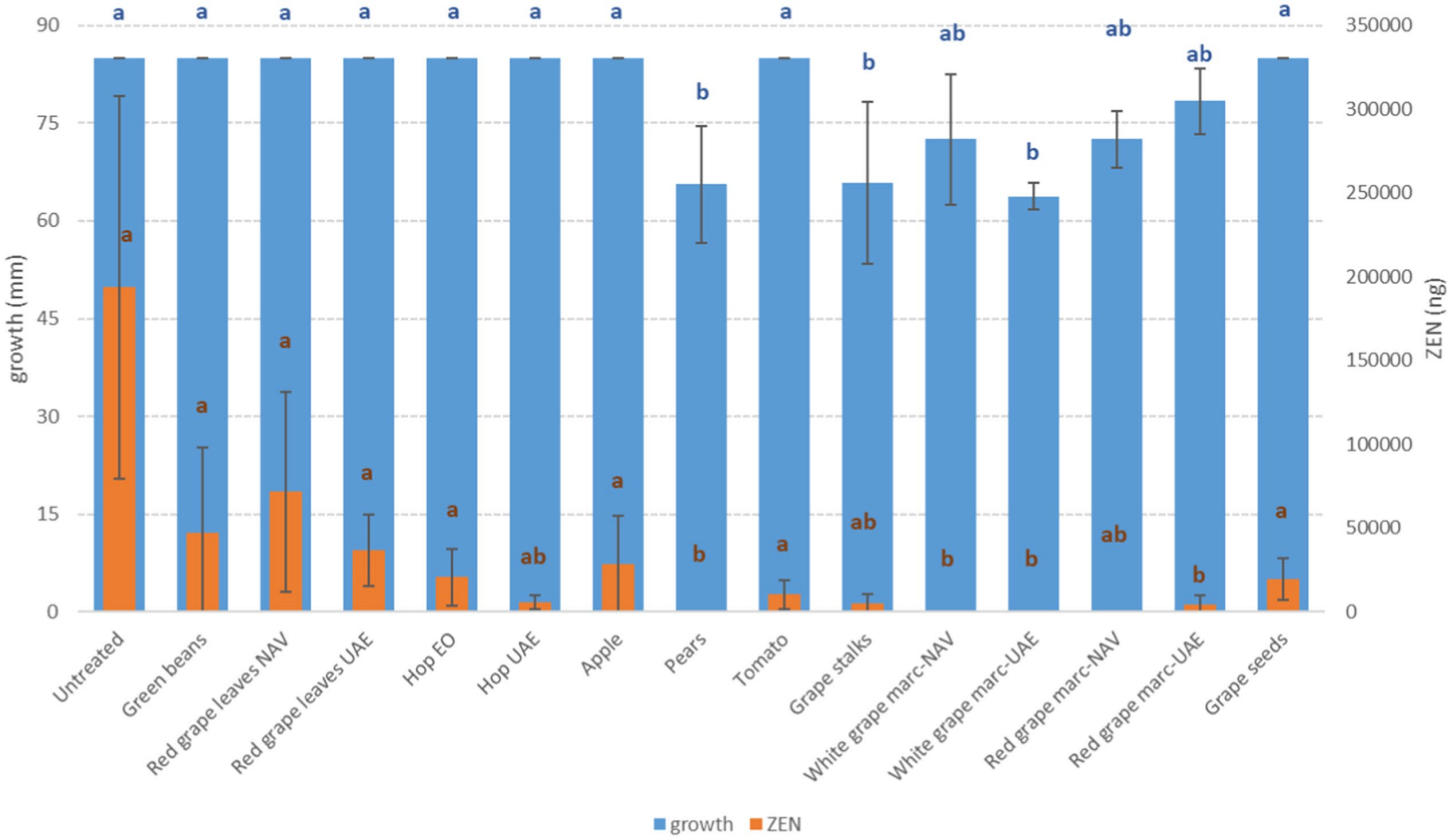
Mean development (mm) and deoxynivalenol production (ZEN, ng) of *F. graminearum* grown on Petri dishes (Ø 90 mm) with potato dextrose agar and 1 ml of different extracts obtained from agricultural production waste at a concentration of 1,000 mg/L after 14 days of incubation at 25°C. Different letters mean significant differences according to Tukey’s test; blue letters refer to significant differences between the theses related to fungal growth, while orange letters refer to significant differences between the theses related to mycotoxin production.

Among the *Fusarium* species considered in this study, more differences in fungal susceptibility were found. While *F. graminearum* was shown to be highly affected in its development by several extracts (GS, PE, and RG-WG) (Figure 3), *F. verticillioides* was only inhibited by PE (−18%) and to a very low and negligible extent by AP (−1%) and GB (−3%) (Figure 4).

**Figure 5:**
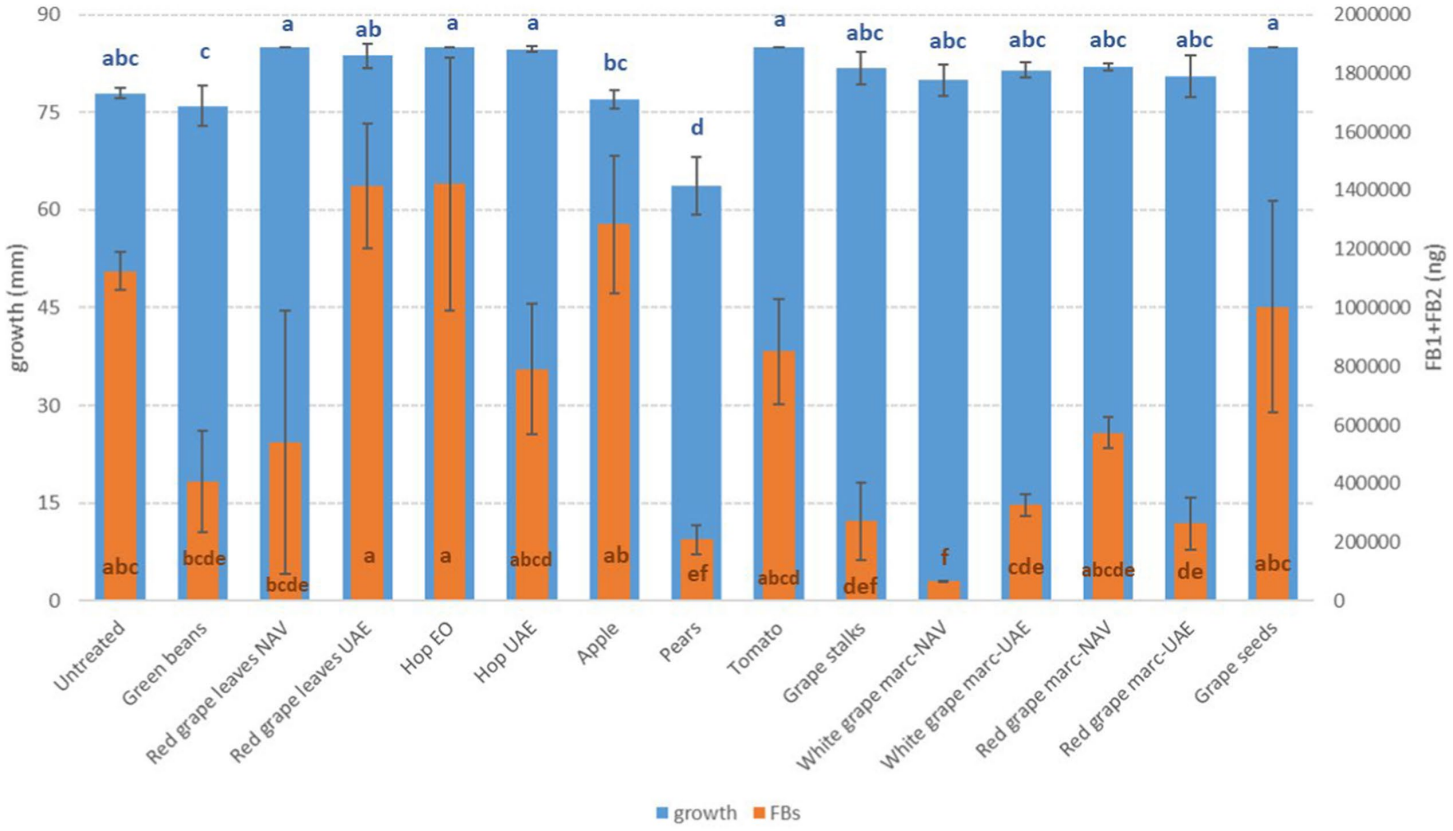
Mean development (mm) and fumonisin production (FB_1_ + FB_2_, ng) of *F. verticillioides* grown on Petri dishes (Ø 90 mm) with potato dextrose agar and 1 ml of different extracts obtained from agricultural production waste at a concentration of 1,000 mg/L after 14 days of incubation at 25°C. Different letters mean significant differences according to Tukey’s test; blue letters refer to significant differences between the theses related to fungal growth, while orange letters refer to significant differences between the theses related to mycotoxin production.

*Alternaria alternata* was less affected by the presence of extracts, resulting in a slight reduction only with GB (−5.8%) (Figure 5).

Interestingly, some extracts were effective against more fungi. In particular, PE extract was one of the most effective against *A. flavus, A. carbonarius, F. verticillioides,* and *F. graminearum*, causing significantly lower development in the considered fungi (*p* ≤ 0.01) (Figures 1–4). Marcs from both red and white grapes can significantly reduce fungal development in all fungi considered, with the sole exception of *A. alternata* and *F. verticillioides* (*p* ≤ 0.01) (Figures 1–3).

### 3.3. Inhibition of mycotoxin production

The production of mycotoxins was more affected than fungal growth. The reductions obtained from the extracts varied from 2.3 to 70.7% for OTA, from 5 to 74.8% for AFB_1_, from 8.5 to 79.7% for DON, from 10.8 to 94% for FBs, from 63 to 100% for ZEN, and from 6.6 to 95.9% for *Alternaria* toxins (Table 6).

It is noteworthy that ZEN and DON produced by *F. graminearum* often showed opposite behaviors when treated with the same extracts. In particular, with RG-WG obtained from NAV, regardless of the grape color, ZEN was completely inhibited, while DON was increased more than 100 times. The same happened with RG obtained from UAE and with PE extracts.

Taking into consideration only the significant differences in mycotoxin production highlighted by ANOVA, species-dependent results were obtained.

Regarding OTA, although good reductions were obtained with several extracts (GS, PE, RG, and RGL UAE), only the essential oil of spent hops was shown to induce a significant reduction in mycotoxin production (*p* ≤ 0.05); on the contrary, AP and VIN induced an increase in OTA production (*p* ≤ 0.05) (Figure 1).

More extracts were able to reduce the production of AFB_1_ by *A. flavus*. In particular, PE extract, RGL UAE extract, and hop essential oil were the most effective in significantly reducing mycotoxin production (*p* ≤ 0.01), obtaining a decrease ranging from 57.7 to 74.8%. However, significant reductions were also obtained by GS, AP, and WG independently of the extraction method (*p* ≤ 0.01), although to a lesser extent (Figure 2).

*Fusarium* species showed completely different behavior. Considering DON produced by *F. graminearum*, a significant reduction in mycotoxin production was obtained only by WG UAE (−72%) (*p* ≤ 0.05), but good reductions were also obtained by TO and AP extracts, with 60 and 30% less production, respectively, although not significant from a statistical point of view (Figure 3).

Regarding ZEN, the other mycotoxin produced by *F. graminearum*, all the RG-WG and PE extracts were the most effective in its reduction (−100%) (*p* ≤ 0.01); satisfactory results were also obtained using HW-UAE (−97%) (Figure 4).

For FB, more extracts were able to significantly reduce the mycotoxins. The most effective were WG and DON, but in this case, they were extracted by NAV and PE extracts with reductions higher than 80% (*p* ≤ 0.01) (Figure 5). The production of FBs by *F. verticillioides* was, however, significantly reduced by approximately 70% when GS and WG were extracted by UAE (*p* ≤ 0.01) (Figure 5).

**Figure 6:**
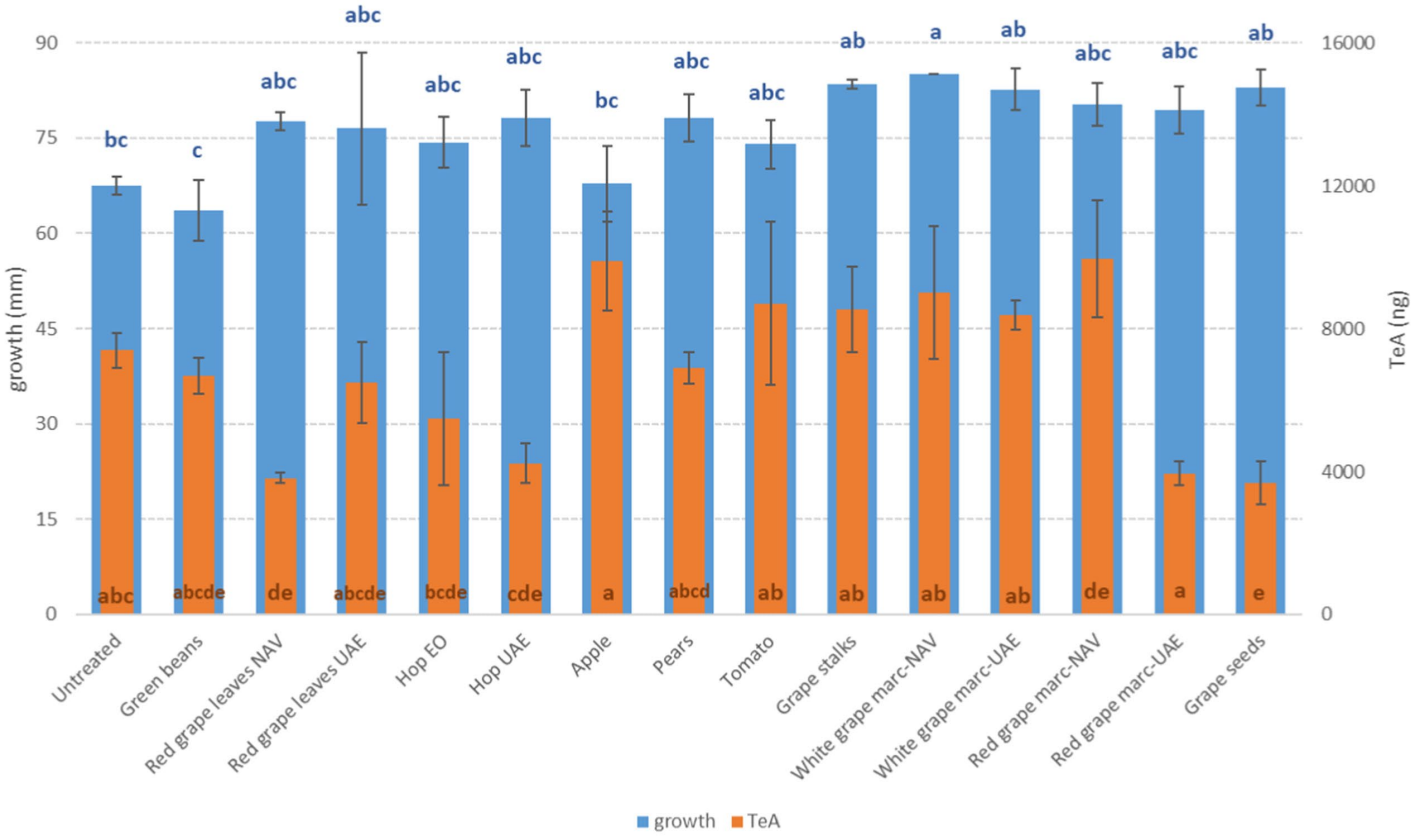
Mean development (mm) and tenuazoic acid production (TeA, ng) of *A. alternata* grown on Petri dishes (Ø 90 mm) with potato dextrose agar and 1 ml of different extracts obtained from agricultural production waste at a concentration of 1,000 mg/L after 14 days of incubation at 25°C. Different letters mean significant differences according to Tukey’s test; blue letters refer to significant differences between the theses related to fungal growth, while orange letters refer to significant differences between the theses related to mycotoxin production.

**Figure 7:**
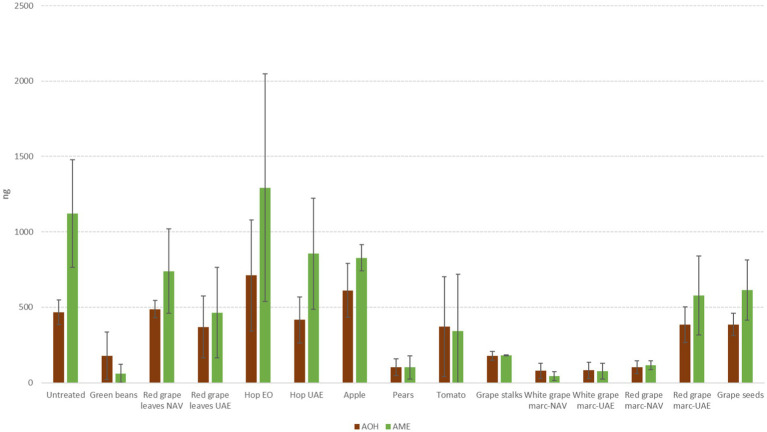
Mean production of alternariol (AOH, ng) and alternariol monoether (AME, ng) of *A. alternata* grown on Petri dishes (Ø 90 mm) with potato dextrose agar and 1 ml of different extracts obtained from agricultural production waste at a concentration of 1,000 mg/L after 14 days of incubation at 25°C.

For *Alternaria* toxins, it is difficult to highlight some extracts that are able to contrast all the mycotoxins considered in the same way. In many cases, we observed a significant reduction for some *Alternaria* toxins and, at the same time, a high production for others among those considered. Taking into account TeA, often the most abundant in cereals, the most significant reductions were obtained with HW- extracted UAE (−42.8%) and with VIN extract (−50.3%) (*p* ≤ 0.01) (Figure 6). For the other mycotoxins considered, the best results were obtained instead by WG, both NAV, and UAE, for AOH (about −82%) (*p* ≤ 0.01) and by GB (−94.4%) and WG-NAV (−95.9%) for AME (*p* ≤ 0.01) (Figure 7). Although not statistically significant for all Alternaria toxins considered, GB, VIN, RGL-UAE, and HW-UAE extracts were able to reduce all three toxins to different extents (Figure 7).

## 4. Discussion

The different results obtained by the different agri-food waste extracts tested in this work were probably due to their varying chemical compositions. Several published studies reported the antifungal activities of pure chemical compounds; some of them also showed interesting results against mycotoxigenic fungi. Compounds belonging to the terpene and benzaldehyde classes showed reducing activities against aflatoxin-producing *Aspergillus* species: o-vanillin damaged the mitochondria of *A. flavus* (35, 36), and some essential oils (cinnamaldehyde, thymol, citral, and carvacrol) inhibited *A. flavus* growth and AFB_1_ production (37). In addition, several organic acids (such as benzoic acid, butyric acid, sorbic acid, hop *α*- and *β*-acids, and phenolic acids), flavonoids, and naphthoquinones have also been shown to reduce *A. flavus* growth and AFB_1_ production (11, 38–41).

In our study, a systemic approach was used to evaluate the potential inhibitory effects of 14 different agri-food waste extracts against multiple mycotoxigenic fungi and related mycotoxins. In particular, the agri-food waste extracts that showed the highest reductions against *Aspergillus* and aflatoxins were hop essential oil, rich in terpenes, red grape leaves, and pear extract, rich in flavonoids and phenolic acids.

Regarding toxigenic *Fusarium* species, isoflavonoids, and phenolic compounds showed activities against *F. graminearum* (14) and trichothecene (13). Green bean extract, containing isoflavonoids such as genistein and daidzein, was able to significantly reduce DON, FBs, and ZEA; pear, white, and red grape extracts, rich in phenolic compounds, reduced FBs production.

Finally, the reduction of OTA by RG-NAV extract can be supported by the inhibition of flavonoids on *A. carbonarius* and OTA reported by Ricelli et al. (42).

It is always very important to consider the different effects that the use of extracts may have on fungal growth and mycotoxin production, which are not always correlated. It is possible to observe how the same extract can reduce fungal development but may not be effective on mycotoxins, and *vice versa*. This is quite normal for mycotoxigenic fungi because of their specific nature. In fact, it is well known that stress conditions, such as those caused by restrictions in development due to fungicides, can induce a higher production of mycotoxins by the same fungus (43–45), even if with scarce growth. On the other hand, mycotoxigenic fungi developing in environments without stress conditions can grow very much and quickly without the necessity to produce mycotoxins (46, 47). Considering extracts, they can have a possible effect on fungal behavior, since they are composed of substances naturally present in the plant substrates attacked by fungi and that can be used, in some cases, as nutritional compounds by some species. For this reason, if extracts are to be used as possible biofungicides, it is important to study their species-specific effects, since for some fungal species they can boost their development (but at the same time create less stress and thus lower mycotoxin content), and for other fungal species the same extract may limit their growth (but at the same time increase mycotoxin content). Our study confirms the findings reported in previous works that evaluated the antifungal properties of some agri-food waste. Spent coffee grounds showed detoxification for AFBs and OTA (11); lemon peel extract, rich in flavonoids, reduced aflatoxin toxicity in rats (48); olive mill wastewater, produced during olive oil extraction, was shown to suppress AFB_1_ produced by *A. flavus*, although no inhibition against fungi was determined (49); apple pomace extracts, rich in phloridzin and quercetin derivatives, showed inhibitory properties against *F. oxysporum* and *N. fischeri* (50). Moreover, application as nano-emulsion or micro-capsulation improved the efficacy of food waste extracts; Faouk et al. (51) reported that nano-emulsion of ginger essential oil through an edible coating increased its bioactivity, achieving higher inhibition of *A. flavus* and related AFB production. Finally, Badr et al. (52) proposed the use of encapsulated grape by-products (stems and leaves) as bio-preservatives in foods; their utilization provided relevant OTA reduction.

However, fewer encouraging results were obtained with some extracts that promoted the growth and production of fungi and related mycotoxins. The PE and WG-NAV extracts, even if they inhibited the growth of *F. graminearum* and the production of ZEN, promoted the production of DON (on average 50% with respect to the control test). In addition, OTA was promoted by AP and VIN extracts, with percentages that exceeded 100%.

## 5. Conclusion

Large amounts of waste and by-products are generated along the whole food chain, which, before being discarded, could be significantly valorized as they represent a source of bioactive compounds. In the present work, high-tech extraction processes with high sustainability were applied to a wide range of organic by-products produced in the agro-food sector in order to obtain extracts rich in polyphenols and terpenes. These extracts demonstrated to be a valuable tool for the *in vitro* inhibition of some of the most important mycotoxigenic fungi and related mycotoxins. These extracts could be applied in the field or during food storage to reduce the mycotoxin contamination of raw materials to regulatory limits. This could significantly reduce the use of chemical pesticides, which are largely used today, with cascading positive effects on the environment, biodiversity, and human health.

## Data availability statement

The raw data supporting the conclusions of this article will be made available by the authors, without undue reservation.

## Author contributions

PG and TB designed and supervised the research. PG, TB, MT, and GL wrote the manuscript. TB, MS, GS, and AG assisted in drafting and revising the manuscript. PG, GB, TB, GL, and MT conducted the trials and analyzed the samples. All authors wrote the manuscript and approved the final version of the manuscript.

## Funding

This research was supported by the 2014–2020 Emilia-Romagna Rural Development Program, Operation Type 16.1.01, Operational Groups of the European Innovation Partnership “Agricultural Productivity and Sustainability,” Focus Area 3A, Innovation Plan “MIlk_COntrollo.”

## Conflict of interest

The authors declare that the research was conducted in the absence of any commercial or financial relationships that could be construed as a potential conflict of interest.

## Publisher’s note

All claims expressed in this article are solely those of the authors and do not necessarily represent those of their affiliated organizations, or those of the publisher, the editors and the reviewers. Any product that may be evaluated in this article, or claim that may be made by its manufacturer, is not guaranteed or endorsed by the publisher.
